# What Vowels Can Tell Us about the Evolution of Music

**DOI:** 10.3389/fpsyg.2017.01581

**Published:** 2017-09-22

**Authors:** Gertraud Fenk-Oczlon

**Affiliations:** Alpen-Adria-Universität Klagenfurt Klagenfurt, Austria

**Keywords:** vowels, timbre, musical scale, non-Western cultures, ethnomusicology, musical protolanguage, evolution of music, linguistic typology

## Abstract

Whether music and language evolved independently of each other or whether both evolved from a common precursor remains a hotly debated topic. We here emphasize the role of vowels in the language-music relationship, arguing for a shared heritage of music and speech. Vowels play a decisive role in generating the sound or sonority of syllables, the main vehicles for transporting prosodic information in speech and singing. Timbre is, beyond question, the primary parameter that allows us to discriminate between different vowels, but vowels also have intrinsic pitch, intensity, and duration. There are striking correspondences between the number of vowels and the number of pitches in musical scales across cultures: an upper limit of roughly 12 elements, a lower limit of 2, and a frequency peak at 5–7 elements. Moreover, there is evidence for correspondences between vowels and scales even in specific cultures, e.g., cultures with three vowels tend to have tritonic scales. We report a match between vowel pitch and musical pitch in meaningless syllables of Alpine yodelers, and highlight the relevance of vocal timbre in the music of many non-Western cultures, in which vocal timbre/vowel timbre and musical melody are often intertwined. Studies showing the pivotal role of vowels and their musical qualities in the ontogeny of language and in infant directed speech, will be used as further arguments supporting the hypothesis that music and speech evolved from a common prosodic precursor, where the vowels exhibited both pitch and timbre variations.

## Introduction

The evolution of music, the evolution of language and possible common evolutionary pathways of these achievements remain a matter of debate. Is music only a non-adaptive by-product of language as suggested by Pinker (1997) or did “previously developed musical powers” (Darwin, 1871, p. 12) precede the oratory? An intriguing hypothesis is that music and language share a common origin in form of a musical protolanguage in which song-like strings had holistic meanings (e.g., Jespersen, 1894; Mithen, 2005; Fitch, 2006).

Here, we emphasize the role of vowels in the music-language relationship^1^, and present arguments that reinforce the hypothesis of a prosodic protolanguage or ‘protomusic’ (Fitch, 2010). Vowels play a decisive role in generating the sound or sonority of syllables, the main vehicles for transporting prosodic information in speech, and singing. In tone languages, which represent more than half the world’s languages, vowels carry the pitch modulations that convey grammatical and lexical information. The tight bond between vowels and pitch is supported by experimental findings suggesting strong interactions in the processing of vowels and melody, but not between consonants and musical information: “Vowels sing but consonants speak” (Kolinsky et al., 2009, p. 1). Likewise, a mismatch negativity (MMN) study by Lidji et al. (2010) revealed a close processing relationship between vowels and pitch even at a pre-attentive level.

This perspective paper starts with a brief theoretical analysis of the sound systems of language and music, highlights general parallels in sound inventories, and reports coincidences between vowel systems and musical scales even in specific cultures. Moreover, we demonstrate a close match between vowel pitch and musical pitch in nonsense syllables of Alpine yodelers and in the yodeling of African Pygmies. We will discuss these findings in the context of other ethnomusicological findings, frequently used (Wiora, 1962; Nikolsky, 2015) to shed light on prehistoric music or on the origin of music. Studies showing the pivotal role of vowels and their musical qualities in the ontogeny of language and in infant directed speech will be used as further arguments for the musical protolanguage hypothesis.

## Sound Systems in Music and Language: Pitch and Timbre

Jackendoff (2009, p. 198) emphasizes the differences in the sound system of language and music: “In phonological structure, the repertoire of speech sounds forms a structured space of timbres /…/. In music, by contrast, the notes are distinguished by the way they form a structured space of pitches…”

But the distinction between timbre contrasts in speech on the one hand and pitch contrasts in music on the other is not always as clear-cut. Especially in some non-Western music, vocal timbre and musical melody are often intertwined. Walker (1997) provides evidence that Canadian Inuit, West Coast Native people, and Australian Aborigines do not distinguish between melody and vocal timbre when discussing their music. In the language of the Inuit, for instance, there does not even exist a word that differentiates between language and music: *nipi* means music and sound of the spoken voice (Nattiez, 1990). And microtonal pitch intervallic movements that could be related to vocal timbre changes (Walker, 1997) are characteristic for Australian Aboriginal music. Vocal timbre and timbral variations are also the main idea behind khasmatonal music whose tonal organization can be characterized as “half-spoken/half-sung with intense timbral/pitch modifications” (Nikolsky, 2015, p. 13). In addition to vowel timbre, which seems to be pivotal in timbre-driven music, a person’s voice timbre (Sheikin, 2002) is an important and often exaggerated element in the music of many Siberian ethnicities.

### Vowels: Timbre, Intrinsic Pitch, Intensity, Duration

Timbre is, beyond question, the primary parameter that allows discriminating between different vowels and to some degree also between different consonants (e.g., nasals, liquids). The human voice, as well as the sounds of most musical instruments, is “made up of many nearly harmonically related frequency components, or partials” (Pierce, 1999, p. 8). The timbre of sounds is determined by the distribution of the sound energy among partials (overtones) of different frequencies.^2^ Vowels differ from each other by specific peaks or formants in their sound spectra, whereas the formants F1 and F2 are most relevant for their identification (Peterson and Barney, 1952). The formants correspond to the resonances of the vocal tract or oral cavity^3^; the main articulatory parameters responsible for vowel timbre are tongue height, front-to back position of the tongue, and lip rounding.

But vowels not only differ in their timbre but also in their intrinsic pitch, intensity, and duration. It is generally postulated that open (low) vowels have a higher intrinsic duration and a higher intrinsic intensity than close (high) vowels (for more details see Möbius, 2003). Concerning vowel intrinsic pitch, it is known since Meyer (1896) that high vowels such as [i] have a higher intrinsic fundamental frequency (IF0) than low vowels such as [a]. While the mechanism which determines IF0 is still a subject of debate, there seems to be general agreement that vowel pitch depends primarily on the frequency of the second formant F2 (Marks, 1978; Traunmüller, 1986). Vowels with a high F2 (e.g., [i], [y]) have a higher intrinsic pitch than vowels with a low F2 (e.g., [a], [o]). The close association between F2 or spectral energy allocation and vowel intrinsic pitch indicates that timbre and intrinsic pitch of vowels are closely interrelated and cannot be separated.

### Vowel Timbre and Musical Melody

In Fenk-Oczlon and Fenk (2009b) we hypothesized that in songs containing strings of meaningless syllables the vowels might be connected to melodic direction in close correspondence to their timbre or intrinsic pitch. We tested this assumption based on all monophonic Alpine yodelers (*n* **=** 15) in Pommer’s (1893) collection. The test revealed a surprisingly uniform pattern: the melody descended in 118 out of 121 [i]→[o] successions and ascended in 132 out of 133 [o]→[i] successions. A similar assumption was tested in Austrian traditional songs, which include successions of nonsense syllables. In 24 out of 26 songs in the Dawidowicz (1980) collection we found the expected coincidence between the vowel [i] and the highest pitch in melody (Fenk-Oczlon and Fenk, 2009a).

A strong relationship between vowel timbre and musical pitch in meaningless syllables is also reported in the yodeling of African Pygmies (Fürniss, 1991; Demolin, 2013): front vowels are associated with high pitch and back vowels with low pitch.

Vowel timbre, moreover, plays a key role in transforming spoken information into whistled languages (Meyer, 2008), in ‘talking khomus’ using the jew’s harp to transmit verbal information in Yakut traditional music (Alexeyev and Spiridon, 2004), as well as in many mnemonic systems for transmitting or representing musical melodies (Hughes, 2000).

## Sound Systems in Language and Music: Parallels in the Inventory Size

Are there parallels in the sound inventory size of language and music? Authors looking for parallels in the sound inventories of language and music often compared the whole phonemic inventory to musical pitches per octave and found that the number of phonemes across languages varies to a much greater extent [“from 11 in Polynesian to 141 in the languages of the Bushmen” (Besson and Schön, 2001, p. 235)] than the number of pitches per octave. And Rakowski (1999) argues that the number of phonemes in languages is much higher than the number of musical intervals, which roughly corresponds with Miller’s magical number seven. An alternative approach was provided in Fenk-Oczlon and Fenk (2009b), comparing only the vowel inventories with the number of intervals in a musical scale, instead of the whole phonemic inventory.

Concerning the vowel inventories across languages there is unanimous consensus that five-vowel systems are the most frequent ones, followed by six and seven vowel-systems (Crothers, 1978; Schwartz et al., 1997; Ladefoged, 2005; Maddieson, 2005). For instance, Maddieson’s (2005) sample in the World Atlas of Language Structures (WALS) comprises 563 languages; the smallest vowel quality inventory is 2 and the largest is 14. Most of the languages have five vowels, followed by six and seven. Four languages have two contrasting vowel qualities, only one language (German) has 14 basic vowels, and only two languages (British English; Bété) make use of 13 vowels. Note, however, that in the respective samples only basic vowel qualities are counted, and variations of basic vowel qualities such as nasalisation, pharyngalisation, length, etc. are not considered.

Unfortunately, statistical databases for scale types across cultures, analogously to those of vowel inventories, do not seem to exist. Musical scales “are classified according to the number of tones used, their range, and their intervals” (Nettl, 1956, p. 46). The simplest scales are diatonic (Nettl, 1956), and Burns (1999) argues that 12 pitches per octave represent a practical limit. The existence of a higher number of tones – e.g., through “intervals that bisect the distance between the Western chromatic intervals” – is, at least as a standard in the culture in question (the Arab-Persian system), a rather controversial question (Burns, 1999, pp 217–218). There is a general agreement that five-tone (pentatonic) scales are the most frequent ones amongst traditional forms of music, and that musical scales across cultures typically have five to seven pitches (Trehub et al., 1999, p. 965). In contrast to language, where six-vowel systems appear to be slightly more frequent than seven-vowel systems, the seven-tone scale (heptatonic scale) seems to be more frequent than the six-tone (hexatonic) scale. Concerning the lower number of six-note scales in comparison to five- or seven-note scales, Gill and Purves (2009) argue that six-note variants of five- or seven-note scales are very frequent e.g., in blues scales, or in melodies using only six out of the seven tones of the heptatonic scale, or using passing tones not included in the pentatonic scale. They assume that six-note scales “are simply not recognized as formally as their five- and seven-note counterparts in Western music theory” (p. 8). In non-Western musical cultures pentatonic scales are, as in Western music, the most frequent ones; but it is interesting to see, that they use, according to Nettl (1956, p. 60), hexatonic scales more frequently than heptatonic scales.

We here state some striking coincidences in the sound inventories of language and music: an upper limit of roughly 12 elements, a lower limit of 2 elements, and a frequency peak at 5–7 elements. It should be noted, that in our comparison between vowels systems and musical sales we only considered the number of elements, and we did not consider the patterning of intervals in a scale or the distribution of vowels in the vowel space.

### Are There Correspondences between Vowels and Scales Even in Specific Cultures?

In Fenk-Oczlon and Fenk (2009a) we speculated that there might be coincidences between vowels and scales even in specific cultures. Consistently, most of the Australian Aboriginal languages have three vowels, only those of the Northern Territory are reported to have more vowels (Butcher and Anderson, 2008). And Lauridsen (1983) reports exactly from those cultures in the Northern Territory the use of a higher number of pitches in song. The most difficult part in testing our assumption is to establish which use of certain musical features is indigenous, and which comes as cultural borrowing. Therefore, looking for coincidences between the number of vowels and number of pitches may be particularly informative in cultures before they had extended contact with other musical traditions. Some more evidence we found so far:

The indigenous cultures of the Americas – such as the Arapaho, Blackfoot, Cheyenne, Comanche, Klamath, Modoc, Navaho, Pawnee – frequently have only four or three vowels (Maddieson, 2005), and tritonic and tetratonic scales are frequently used in their music (Rhodes, 1954; Nettl, 1956). Noteworthy, Nettl (1956, p. 112) reports that in Pueblo music which includes the music of the Hopi, Zuni, and Taos “the scales tend to have more tones than they do elsewhere on the continent.” And Hopi, Zuni, and Taos belong exactly to those rather rare indigenous languages of the Americas which have five or six vowels.

Correspondences between three-vowel systems and tritonic scales show in the Inca musical tradition and in the music of Greenland. Pre-Hispanic herranza ritual music of the Andes is generally tritonic –rather than “incomplete pentatonic” (Holzmann, 1980), and Incan Quechua as well as other Quechuan varieties have just three vowels. Greenlandic like the vast majority of Inuit languages has only three vowels, and tritonic modes are still common in the music of East Greenland (Olsen, 1972). Moreover, tritonic patterns are supposed to have been characteristic to Inuits from Greenland to Siberia.

The correspondences found between vowel inventories and musical scales are, of course, highly tentative. Nevertheless, it would be interesting to investigate whether these correspondences can also be found in other autochtonous cultures.

## Vowels in Ontogeny, Phylogeny, and Motherese

Vowels and their musical qualities play a pivotal role in the ontogeny of language and in infant directed speech. Vowel-like sounds are the first speech sounds that children produce and are already present in the cooing stage appearing at about 6–8 weeks of age. “The first coos that infants make sound like one long vowel” and the infant’s learning process to be undertaken in this stage is to “produce a series of different vowel-like sounds strung together but separated by intake of breath” (Hoff, 2009, p. 143). The coos exhibit pitch variations, which according to Halliday (1975, cited in Masataka, 2007) are already connected with pragmatic functions. For instance: requests for objects are associated with a rising pitch, and labeling is associated with falling pitch. Masataka (2007) argues that pitch variations of this sort might be strengthened by caregivers in using infant directed speech or motherese, which can be above all characterized by high pitches and exaggerated pitch contours. Thus, infant directed speech is generally considered having many musical qualities and providing a scaffold for language acquisition (Falk, 2004). Again it is the vowels that are acoustically exaggerated and the more caregivers are exaggerating the vowels, and the larger the vowel space they use, the better the infant’s language performance (Liu et al., 2003). The importance of vowel pitch or vowel timbre in early communication is demonstrated by Papousek’s (1992) findings (cited in Walker, 1997) where infants’ abilities to discriminate pitch variations in the mother’s voice is linked to “vowel” pitch, not “musical” pitch.

But vowels or vowel-like sounds may not only play a prominent role in the ontogeny of human language, but also in its early evolution. Notably, recent work by Boë et al. (2017) demonstrated astonishing similarities between human vowels and “vowel like segments” produced by Guinea baboons. Combining acoustical analyses of baboon vocalizations with an anatomical study of the animals’ vocal tract, the authors showed that baboons are capable to produce and combine five vowel-like sounds, despite their high larynx, suggesting a proto-vocalic system was already present in the last common ancestor of humans and baboons, at least 25 million years ago (Boë et al., 2017).

## Discussion and Conclusion

We started with a theoretical analysis of the sound systems of language and music and and demonstrated a closer relationship and more parallels between these systems than generally assumed. Vowels show all the core properties of music, i.e., timbre, intrinsic pitch, intensity, and duration. We revealed general correspondences between vowel systems and musical scales across cultures, and presented correspondences even in specific cultures. While the general parallels in the sound inventories may reflect cognitive and physiological constraints of our auditory and articulatory apparatus, the coincidences found between number of vowels and number of tones in specific cultures indicate a tight bond between vowels and music. The match between vowel pitch and musical pitch in meaningless syllables of Alpine yodelers and yodelers of African Pygmies and the relevance of vocal timbre in the music of many non-Western cultures, in which vocal timbre/vowel timbre and musical melody are often intertwined, further support a close relationship between vowels and music. How can these findings be related to the evolution of music?

The evidence we provided for a close relationship between vowels and musical pitch stems from studies of ethnic or non-Western music. And ethnomusicological findings are often assumed to particularly revealing with regards to the origin of music. For example, Nikolsky (2015) uses ethnic music to reconstruct prehistoric music and assumes that the earliest music “was organized not by pitch, but by timbre” (p. 25). This assumption implicates that at this early stage, music and speech must have been together and that it was the vowels which displayed – besides voice timbre – timbre and pitch modulations.

Taking further into account the pivotal role of vowels in ontogeny – phylogeny in some respects and to some degree resembles ontogeny and vowel-like sounds are the first speech sounds that children produce – it is tempting to speculate that the earliest human vocal communication started with vowels or vowel syllables strung together, which were connected by semivowels or glides such as [w], [h], [j] or the glottal stop [Ɂ]. The sequences of vowels exhibited pitch and timbre modulations, which were combined for different pragmatic and social functions, and were probably propositionally meaningless (cf. also Fitch, 2010). In a later stage, more ‘real’ consonants such as obstruents emerged and were combined with vowels into consonant-vowel syllables. This was likely the emergence of articulated speech (Jordania, 2006) and of utterances which could express propositional meanings.

We still encounter vocal music in which mere vowel sounds are connected. For example, Lewis (2013) reports that the songs of the BaYaka Pygmies rarely have words, but many songs are based on vowel-sound melodies or hocketed vowel sounds.

In conclusion, we have demonstrated a close relationship between vowels and music in non-Western cultures which may shed light on the earliest human vocal communication and may strengthen the idea of a musical protolanguage. Although our findings are preliminary, we are convinced that future research combining ethnomusicological findings with those from linguistic typology will provide further insight into the evolution of music, and in music as such.

## Author Contributions

The author confirms being the sole contributor of this work and approved it for publication.

## Conflict of Interest Statement

The author declares that the research was conducted in the absence of any commercial or financial relationships that could be construed as a potential conflict of interest.

## Footnotes

1 There are, of course, many other areas where music and language show similarities. For example, striking rhythmic relationships between music and speech (Patel and Daniele, 2003; Neuhoff and Lidji, 2014) or correspondences in the length of ‘intonation units’ (Fenk-Oczlon and Fenk, 2009b; Lehmann and Goldhahn, 2016).

2 In fact, timbre does not only depend on spectral aspects of sound but also on time-varying aspects, i.e., the amplitude envelope (e.g., Caclin et al., 2005).

3 For a detailed description of the human vocal tract, its relationship to vowel formant frequencies and potential relevance in the evolution of human language (see, e.g., Fitch, 2010), and a recent discussion of this interesting topic by Fitch et al. (2017) and Lieberman (2017).
